# The treatment strategy of R0 resection in colorectal cancer with synchronous para-aortic lymph node metastasis

**DOI:** 10.1186/s12957-020-02007-2

**Published:** 2020-08-28

**Authors:** Hajime Ushigome, Masayoshi Yasui, Masayuki Ohue, Naoaki Haraguchi, Junichi Nishimura, Keijirou Sugimura, Kazuyoshi Yamamoto, Hiroshi Wada, Hidenori Takahashi, Takeshi Omori, Hiroshi Miyata, Shuji Takiguchi

**Affiliations:** 1 Department of Gastroenterological Surgery, Osaka International Cancer Institute, 3-1-69 Otemae, Chuo-ku, Osaka, 541-8567 Japan; 2 Department of Gastroenterological Surgery, Nagoya City University, Nagoya City, Japan

**Keywords:** Para-aortic lymph node metastasis, Synchronous, Colorectal cancer

## Abstract

**Background:**

Synchronous metastatic para-aortic lymph node (mPALN) dissectionin colorectal cancer has relatively good oncological outcomes, though many patients develop recurrence. Universal prognostic factor remain unclear and no definitive perioperative chemotherapy is available, making the treatment of mPALN controversial. In the present study, we aimed to establish a treatment strategy for synchronous mPALN.

**Methods:**

This retrospective study involved 20 patients with pathological mPALN below the renal vein who underwent R0 resection. Long-term outcomes, recurrence type, and prognostic factors for survival were investigated.

**Results:**

The 5-year overall survival and recurrence-free survival rates were 39% and 25%, respectively. Seventeen patients (85%) developed recurrence, including 13 (76%) within 1 year after surgery, and ~ 70% of all recurrences were multiple recurrences. Four patients (20%) survived > 5 years. Pathological T stage (*p*= 0.011), time to recurrence (*p* = 0.007), and recurrence resection (*p* = 0.009) were identified as prognostic factors for long-term survival.

**Conclusions:**

R0 resection of synchronous mPALN in colorectal cancer resulted in acceptable oncological outcomes, though we found a high rate of early unresectable recurrence. If the recurrence occurs late or isolated, surgical resection should be considered for longer survival.

## Background

In colorectal cancer (CRC), the relatively rare occurrence of metastatic para-aortic lymph node (PALN) is categorized as stage IV disease [1, 2]. The reported incidence of isolated metastatic PALN is 1–2% [3, 4]. In stage IV patients, R0 resection is usually recommended for resectable liver or lung metastases [2, 5–7]. However, metastatic PALN remains highly controversial as no multicenter studies or randomized controlled trials have been performed on synchronous or metachronous PALN dissection (PALND).

A few studies have been published on metachronous metastatic PALN [8–11], and studies on synchronous metastatic PALN have shown that PALND below the renal veins can be performed safely, prolonging prognosis compared to non-surgical resection [12, 13]. Bae et al. [12] also demonstrated that PALND results in survival rates comparable to synchronous liver metastasectomy. However, although the 5-year overall survival (OS) rates for patients who underwent synchronous PALND reached 25–53%, approximately 80% of the patients experience recurrence [4, 12–15] and few studies have discussed the recurrence pattern [12] or treatment after recurrence. Furthermore, no definitive perioperative chemotherapy has been established.

Recently, three retrospective studies have shown updated prognostic factors for synchronous PALND, including R0 resection, histological type, number of PALN, other distant metastases, metastatic lateral pelvic lymph node, and preoperative CEA values [4, 15, 16]. However, the prognostic factors for synchronous PALND identified in the previous studies are not yet universal.

These findings indicate that the treatment strategy for PALN remains highly controversial.

The aim of this study was to examine the prognostic factors, recurrence patterns, and long-term outcomes in CRC with synchronous metastatic PALN to establish the strategy for PALND.

## Materials and methods

This was a single-center retrospective cohort study at Osaka International Cancer Institute, Osaka, Japan. The study protocol was approved by the institutional review board (No. 18033-2). Written informed consent was waived because of the retrospective design.

### Study population

Between September 1998 and December 2018, we reviewed the medical records of patients who underwent PALND for clinical metastatic PALN in addition to resection of the primary CRC. Twenty-two patients had pathological metastatic PALN. Two patients who could not undergo R0 resection were excluded. R0 resection was defined as having no macroscopic or microscopic residual tumor. A total of 20 patients were included in the present study.

### Indication for PALND

Tumors were pathologically diagnosed as CRC and classified according to the criteria of the World Health Organization and Union for International Cancer Control, 8th edition [17]. Clinical stage was assessed based on enhanced computed tomography (CT) and positron emission tomography (PET). Surgical treatment with CRC resection and PALND was performed based on the following criteria: metastatic PALN diagnosed as clinically positive by the colorectal surgery team and at least two radiologists based on PALN with a long-axis diameter ≥ 7 mm by CT and/or a high intensity spot on PET; metastatic PALN located only below the renal vein; and expectation of R0 resection, including synchronous metastasectomy or second stage metastasectomy, such as metachronous hepatectomy.

### PALND

PALND was performed after primary tumor resection. PALND was defined as a dissection of all lymphatic and connective tissues around the abdominal aorta and inferior vena cava between the left renal vein and bifurcation of the iliac artery. If lymph node dissection was not performed in this area, it was defined as lymphadenectomy.

### Follow-up

After surgery, all patients underwent general follow-up with examination of tumor markers every 3 months, as well as chest and abdominal CT every 6 months. A colonoscopy was performed every year for rectal cancer, and every other year for colon cancer. If recurrence was suspected during the follow-up, CT and/or PET was added immediately. Local recurrence was defined as recurrence within the pelvic cavity for rectal cancer or around the tumor area for colon cancer.

### Statistical analysis

Data are presented as medians and ranges. The rates of OS and recurrence-free survival (RFS) were calculated using the Kaplan–Meier method, and the log-rank test was used to compare OS between the two groups. *P* < 0.05 was considered significant. Statistical analyses were performed using JMP version 8.0.2 software (SAS Institute, Cary, NC).

## Results

### Clinicopathological characteristics

The demographic and pathological characteristics of the 20 patients are given in Table 1.

**Table 1:**
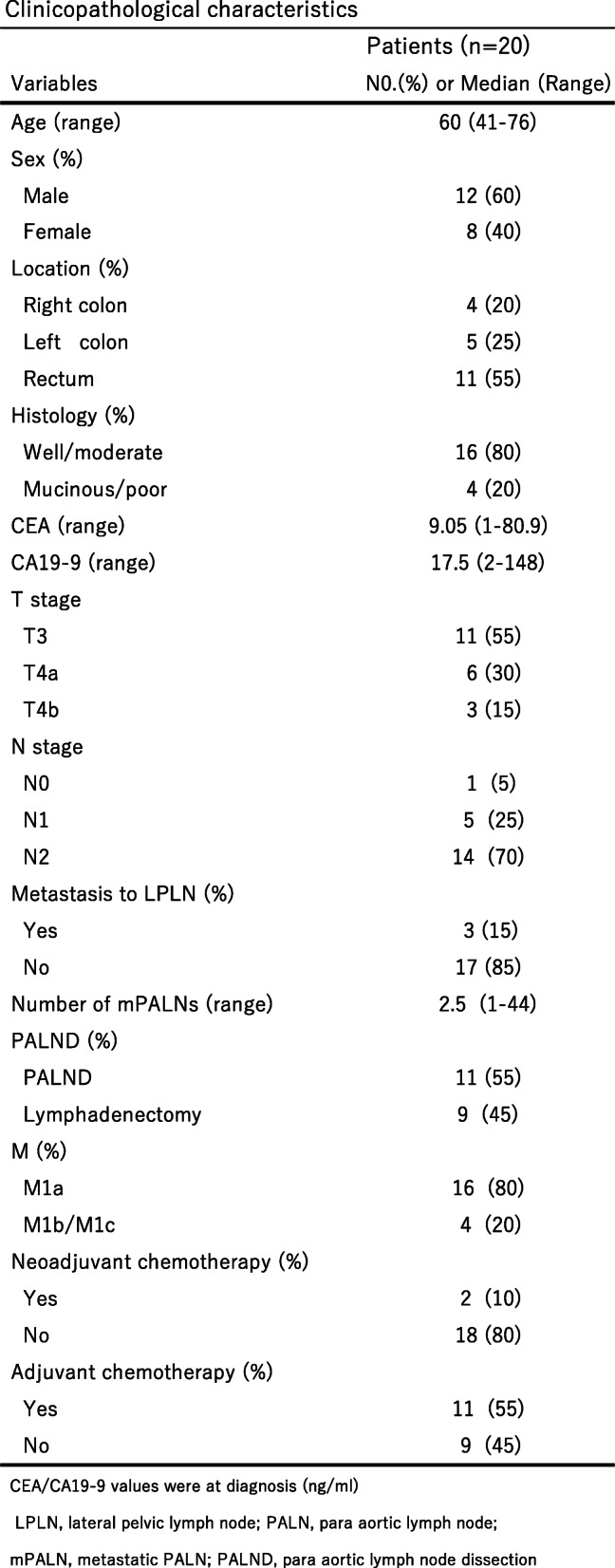
Clinicopathological characteristics

CEA/CA19-9 values were at diagnosis (ng/ml)

LPLN, lateral pelvic lymph node; PALN, para aortic lymph node; mPALN, metastatic PALN; PALND, para aortic lymph node dissection

Except for metastatic PALN, distant metastasis was confirmed in 4 patients (liver metastasis, *n* = 2; ovarian metastasis, *n* = 1; peritoneal dissemination, *n* = 1), all of which were resected synchronously. Adjuvant chemotherapy (AC) was performed in 12 patients (60%), 9 (75%) with camptothecin-11 or oxaliplatin, whereas neoadjuvant chemotherapy with oxaliplatin (NAC) was used in only 2 patients (10%).

### Long-term outcomes and recurrence

**Fig. 1:**
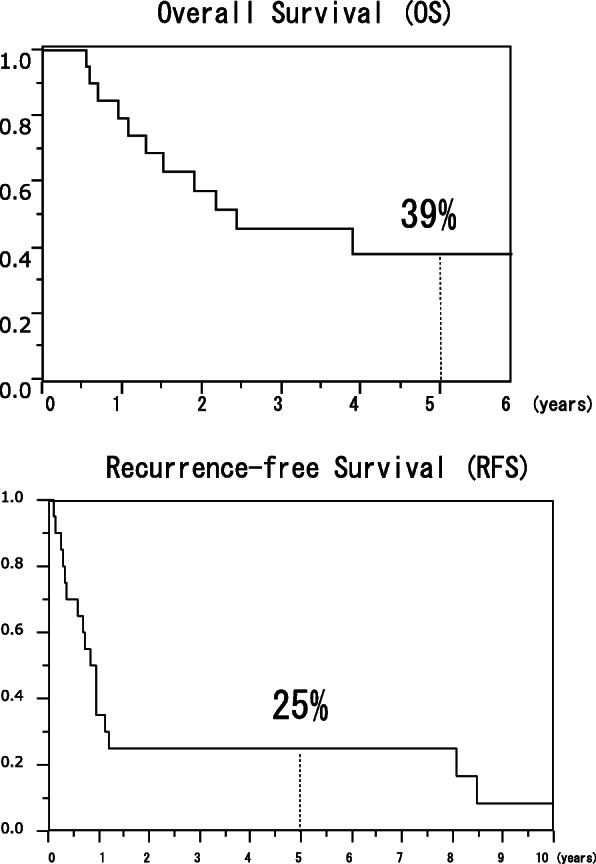
Overall survival and recurrence-free survival for the patient with R0 PALND

The median follow-up duration was 24.8 months (6.6–248.1). The 5-year OS was 39% and 5-year RFS 25% (Fig. 1). Seventeen patients (85%) developed recurrence, including two cases of recurrence 8 years after surgery. Table 2 showed the pattern of first recurrence in the 17 patients.

**Table 2:**
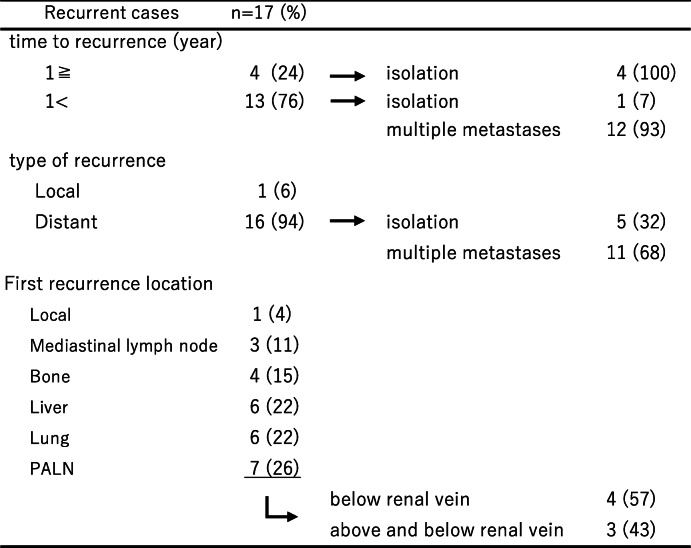
Recurrence pattern after PALND

Thirteen patients (76%) developed recurrence within 1 year after surgery. Almost all of these patients developed distant multiple recurrences that could not undergo surgical resection. However, four of the patients who developed recurrence 1 year after surgery had isolated recurrence, and three underwent surgical resection. The most common recurrence site was PALN (7/27, 26%), and three of the patients developed recurrence both above the renal vein and below the renal vein. Most patients who developed recurrent PALN also had other distant metastases, but one patient developed only isolated PALN and was resected.

### Long-term and short-term survivors

Table 3 shows the clinicopathological findings of four long-term survivors over 5 years and four short-term survivors within 1 year.

**Table 3:**
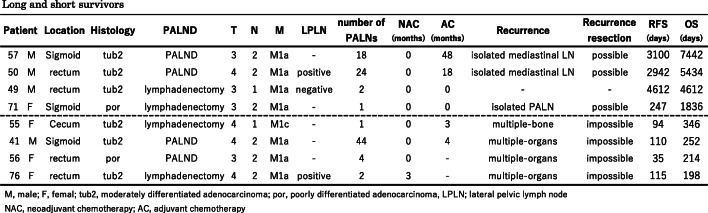
Long and short survivors

M, male; F, female; tub2, moderately differentiated tubular adenocarcinoma; por, poorly differentiated adenocarcinoma, LPLN; lateral pelvic lymph node

NAC, neoadjuvant chemotherapy; AC, adjuvant chemotherapy

All short-term survivors developed early and multiple recurrences with no surgical indication. Two patients developed recurrence during AC, and the other two presented with recurrence before the introduction of AC. Long-term survivors also developed recurrence (3/4, 75%), but most were delayed and all were isolated recurrence that could be dissected curatively. Two patients who developed delayed recurrence had received AC for a long time.

### Prognostic factors in patients with R0 PALND

Pathological T stage (*p* = 0.011), time to recurrence (*p* = 0.007), and recurrence resection (*p* = 0.009) were identified as prognostic factors (Table 4).

**Table 4:**
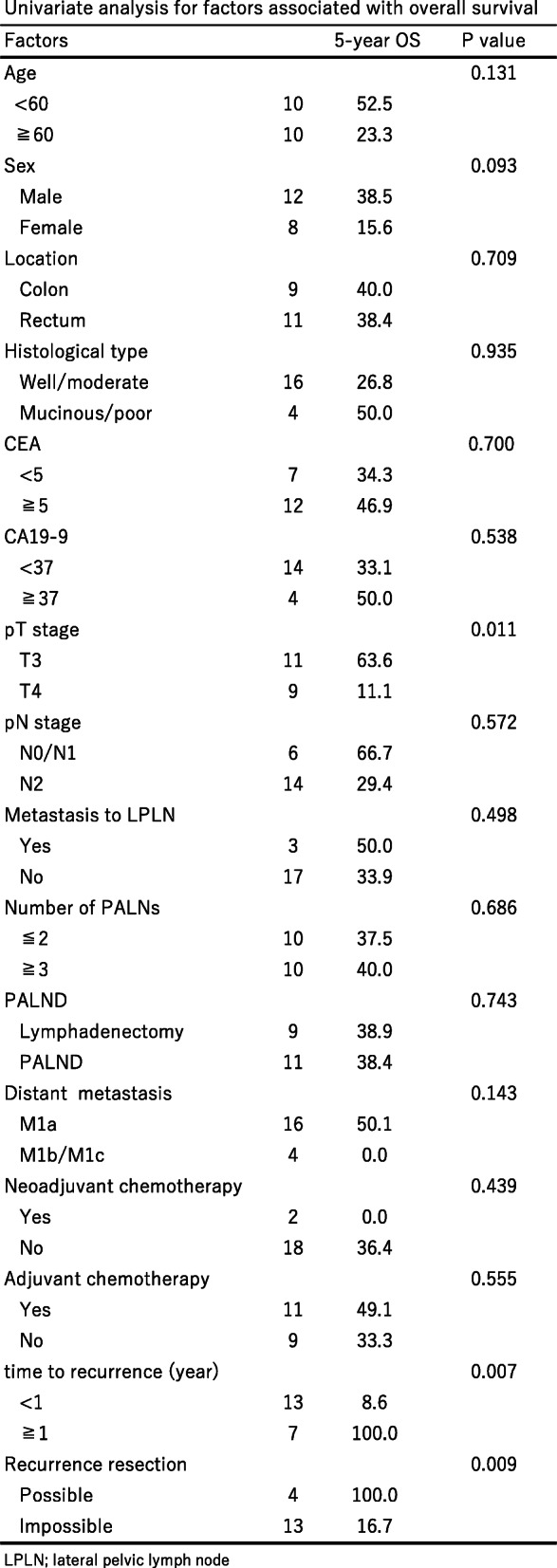
Univariate analysis for factors associated with overall survival

*LPLN* lateral pelvic lymph node

The 5-year OS in the four patients who could undergo resection of recurrence (metastatic PALN, *n* = 1; metastatic mediastinal LN, *n* = 3) was 100%. The number of metastatic PALNs (*p* = 0.686) and PALND (*p* = 0.743) were not identified as prognostic factors. Distant metastasis (*p* = 0.143) was not identified as a risk factor, but no patients who had other distant metastases survived 5 years.

## Discussion

This study was conducted to examine the oncological outcomes in CRC with synchronous PALN to establish the strategy for PALND. We found that PALND is associated with the potential for long-term survival, but also early unresectable recurrences. Pathological T stage, time to recurrence, and recurrence resection were prognostic factors for long-term survival. No previous reports have discussed recurrence, including the time to recurrence and site of recurrence, but the current findings support treatment strategies for patients with synchronous metastatic PALN in CRC.

Our study was limited to patients who underwent R0 resection based on previous reports that R0 resection has a better prognosis than R1–2 resection [14, 15]. The 5-year OS and RFS rates in 20 patients with synchronous metastatic PALN who underwent PALND were 39% and 25%, respectively. These oncological outcomes are similar to what was reported in previous studies [4, 12–15], and relatively more favorable than general stage IV CRC (5-year OS 13.2 to 22 %) [14], which indicates that PALND can prolong prognosis.

In terms of recurrence, 55% of patients received AC, but the overall recurrence rate after PALND reached 85%, and most cases were early and multiple recurrences. Although four patients with isolated recurrences after PALND could undergo resection and experienced long-term survival [18], some patients developed unresectable recurrences before or during AC and died within 1 year after PALND. These findings show that a number of patients who underwent PALND had no indication for PALND in the first place.

We found that pathological T stage, time to recurrence, and recurrence resection are prognostic factors in patients undergoing R0 PALND, though these factors were only known postoperatively and not previously reported. First, though pathological T stage is common as a prognostic factor and useful in determining chemotherapy after resection of CRC without distant metastases [2, 7], in CRC with metastatic PALN, it may be not useful for considering the treatment strategy. We also showed a relationship between recurrence more than 1 year after PALND and long-term survival. Many of the recurrences 1 year after PALND were isolated recurrences, so a longer time to recurrence was more likely to be oncologically favorable. Therefore, it is reasonable that a long time to recurrence was associated with long-term survival from a biological perspective [19].

Resection of recurrence, especially after PALND, has been reported rarely and is highly controversial. Though resection of recurrence was performed in our study, all recurrences were isolated to distant lymph node, and most recurrences occurred more than 1 year after PALND. Thus, the indication for surgical resection was limited. However, considering the number of previous reports reporting prolonged prognosis with surgical resection for lymph node recurrence or repeat hepatic resection for liver re-recurrence [3, 10, 11, 20, 21], if the recurrence is resectable and not early, it would be valuable to perform resection for long-term survival, even after PALND.

In regards to other prognostic factors, distant metastasis and PALND did not have significant differences. However, the M1b/M1c patient did not achieve long survival, and distant metastasis has potential as a prognostic factor [4, 14]. Though PALND was a statistically negative prognostic factor, it should be performed, if possible, because one patient after lymphadenectomy developed PALN recurrence and additional PALND provided long-term survival. Even with our results, universal prognostic factors for synchronous PALND have been unclear and at least a retrospective multi-institutional study is necessary.

Considering the treatment strategy for synchronous PALN, AC was a statistically negative prognostic factor for OS in the present study, though 75% of patients received intensive AC (camptothecin-11 or oxaliplatin), but two patients who developed delayed recurrence had received AC for a long time, indicating that AC had the potential to improve RFS. Although Nakai et al. [14] showed that AC is a prognostic factor after PALND, 94% of patients in their study underwent AC and requires careful interpretation. To interpret our results, it may be reasonable to refer to the previous randomized control study that showed AC (Uracil-tegafur and leucovorin) prolonged RFS, but not OS, after resection of liver metastasis [22].

Our study included only two patients who received NAC, and it was difficult to investigate NAC. However, considering that some oncologically poor patients developed unresectable early recurrences and few universal prognostic factors were available preoperatively, it may be reasonable to perform NAC. Though some studies have shown the efficiency of NAC for stage IV CRC [9, 20], there is little evidence for NAC in patients with metastatic PALN and further investigation is needed.

Some limitations must be considered when interpreting the results of this study. First, this study was a single-center, retrospective analysis. Second, this study covered 20 years and the background for treatment was different, especially chemotherapy. Third, this study had a selection bias, involving only patients with pathological PALN, but did not include the patients who underwent chemotherapy without PALND; therefore, not all PALN-positive patients were included.

## Conclusions

Our results show that R0 synchronous PALND in CRC had relatively good oncological outcomes, and pathological T stage, time to recurrence, and recurrence resection were prognostic factors for PALND. Our results also revealed a high rate of early unresectable recurrences after PALND, but recurrence resection should be considered for longer survival in isolated late recurrence.

## Supplementary information

**Additional file 1.**

## Data Availability

The datasets used and analyzed in this study are not publicly available (to maintain privacy) but are available from the corresponding author on reasonable request.

## Abbreviations

mPALN: Metastatic para-aortic lymph node
CRC: Colorectal cancer
PALN: Para-aortic lymph node
PALND: Para-aortic lymph node dissection
OS: Overall survival
CT: Computed tomography
PET: Positron emission tomography
RFS: Recurrence-free survival
AC: Adjuvant chemotherapy
NAC: Neoadjuvant chemotherapy
LPLN: Lateral pelvic lymph node
